# The significance of the alteration of 8-OHdG in serous ovarian carcinoma

**DOI:** 10.1186/1757-2215-6-74

**Published:** 2013-10-29

**Authors:** Xia Xu, Yan Wang, Wenwen Guo, Yiqing Zhou, Chunmei Lv, Xiaoxiang Chen, Kaijiang Liu

**Affiliations:** 1 Department of Chemotherapy, Jiangsu Institute of Cancer Research, Nanjing, China; 2 Department of Pathology, the Second Affiliated Hospital of Nanjing Medical University, Nanjing, China; 3 Department of Medical Genetics, Nanjing University School of Medicine, Nanjing, China; 4 Jiangsu Key Laboratory of Molecular Medicine, Nanjing University School of Medicine, Nanjing, China; 5 Department of Radiotherapy, Jiangsu Institute of Cancer Research, Nanjing, China; 6 Department of Gynecologic oncology, Jiangsu Institute of Cancer Research, Nanjing, China; 7 State Key Laboratory of Bioelectronics, Southeast University Nanjing, China; 8 Department of Obstetrics and Gynecology, Renji Hospital, Shanghai Jiaotong University School of Medicine, Shanghai, 200127, China

**Keywords:** *hOGG1* gene, 8-OHdG, Epithelial ovarian cancer, Serous carcinoma, p53

## Abstract

**Background:**

Oxidative damage and DNA repair dysfunction are associated with carcinogenesis. 8-OHdG is one of the major oxidative DNA adducts. Present work aims to investigate whether the expression of 8-OHdG and its key repair gene *hOGG1* play distinctive role in two types of serous ovarian cancer.

**Materials and methods:**

8-OHdG level in DNA from tumor and matched tumor-adjacent normal tissue in 48 high-grade papillary serous carcinomas (HG-SOC), 24 low-grade papillary serous carcinomas (LG-SOC), 20 serous cystadenomas, and 16 non-tumor control ovaries was tested. The Cox proportional hazards model and the log-rank test were used to assess the associations between the 8-OHdG level in two types of serous cancer and patients’ survival. Real-time polymerase chain reaction and protein immunoblot were employed to detect *hOGG1* mRNA and protein levels in tumor and adjacent normal tissues. Immunohistochemistry was used to determine the expression of hOGG1 and p53.

**Results:**

There was no difference of average 8-OHdG/10^6^dG DNA level either between HG-SOC (27.8 ± 8.9), LG-SOC (25.2 ± 7.4) and benign serous cystadenoma (26.5 ± 7.7, p = 0.35); or between the tumor-adjacent normal tissue of HG-SOC (18.8 ± 5.2), LG-SOC (21.4 ± 6.5), benign serous cystadenoma (20.5 ± 9.1) and non-tumor ovary (21.6 ± 4.9, p = 0.62). The 8-OHdG/10^6^dG level was significantly higher in tumor comparing to that in matched normal tissue adjacent to carcinoma in HG-SOC (1.52 ± 0.52, p = 0.02), but not in LG-SOC or benign serous cystadenoma. Increased level of 8-OHdG in tumor DNA was an independent factor of overall survival in serous ovarian carcinoma upon multivariate analysis (p < 0.01). Increased level of 8-OHdG in tumor DNA indicates poorer overall and progression-free survival durations than counterparts (47.3 *vs* 105.7 months and 13.5 *vs* 45.3 months, respectively). Protein levels of hOGG1 were remarkably decreased in HG-SOC (p < 0.01), but not in LG-SOC and serous cystadenoma compared with the tissue adjacent to carcinoma. A positive result on p53 immunostaining was associated with lower hOGG1 expression in HG-SOC (p = 0.04).

**Conclusion:**

Increased 8-OHdG level and decreased expression of hOGG1 in tumor were found in HG-SOC but not LG-SOC. Increased 8-OHdG level in tumor DNA was significantly associated with poorer overall survival and progression-free survival in serous ovarian carcinoma.

## Background

Epithelial ovarian cancer (EOC) is the most lethal gynecological malignancy in USA, and the second rank in China while the incidence rate of EOC is evidently increasing during the past decades [1,2]. The fact that the more aggressive high-grade subgroup accounts for more than 50% of EOCs is regarded as the major reason for the poor survival rate of this disease [3-5]. A recent defined model revealed that high-grade serous ovarian carcinoma (HG-SOC) can progress through greater genetic instability that leads to rapid metastasis without an identifiable precursor lesion aside from a stepwise mutation process in low-grade serous ovarian carcinoma (LG-SOC) [6]. HG-SOC was thought to be driven predominantly by multiple amplification and deletion; and nearly all cases have inactivating mutations of p53 [7], whereas 40% exhibit mutations of BRCA1 or BRCA2 [8], while LG-SOC has few copy number abnormalities, but it frequently exhibits activating mutations of Ras, Raf, and PTEN.

Defects in response to DNA damage was one of the central pathogeneses of human malignancies [9]. BRCA1 and BRCA2, involved in DNA repair and cellular response to DNA damage, were reported to be responsible for approximately 5% to 10% of EOCs in patients with a family history [10]. However, whether other DNA repair-associated genes confer risk to the development of sporadic EOC remains uncertain. The genetic variation in base excision repair (BER) system-induced genetic instability linked to malignancy susceptibility is substantial at present [11,12]. The 8-hydroxy-2′-deoxyguanosine (8-OHdG) is one of the best-characterized oxidized bases. 8-OHdG in DNA could lead to mis-incorporation of adenines opposite the 8-OHdG lesion thus inducing G:C to T:A mutations in genomic DNA. 8-oxoguanine DNA glycosylase 1 (OGG1), a bifunctional glycosylase, is mainly involved in repairing 8-OHdG from oxidative damage. Polymorphisms and loss of heterozygozity of *hOGG1* gene, as susceptibility factors for sporadic EOC, have been revealed in several case–control studies [13-16]. In our previous study, we found that variations in the 5′UTR of *hOGG1* gene conferred risk to type II but not to type I EOC [17]. We also suggested that *hOGG1* variations are more frequent in p53 positive breast and ovarian cancer [18,19].

Studies revealed that increasing 8-OHdG level is associated with higher stage and non-optimal surgical outcome in EOC [20,21], and may confer risk to high grade subtype. Comprehensive mapping of TP53 mutation rates in a homogeneous group of EOC patients revealed that mutant TP53 is a driver mutation in HG-SOC pathogenesis [22]. However, there was still no evidence exploring the relationship between 8-OHdG or hOGG1 alteration and the p53 mutation in serous ovarian cancer.

In the present study, we explored 8-OHdG level and compared *hOGG1* mRNA and protein levels in the two types of SOC, benign serous cystadenomas, and normal ovaries (non-tumor control). We also analyzed the relationship between 8-OHdG expression and the survival in serous ovarian cancer. To eliminate differences in base repair ability among individuals, a match pair study of tumor and adjacent non-tumor tissue was conducted.

## Materials and methods

### Subjects and samples

All patients were clinically diagnosed sporadic ovarian tumor and underwent primary treatment in the Department of Gynecologic Oncology, Jiangsu Institute of Cancer Research (JICR), the University of Nanjing Medical University between January 2003 and December 2010. The exclusion criterions were summarized as below: histology confirmed to be other than ovarian tumor; a history of prior cancer, diabetes; an inability to give informed consent, or was either pregnant or breastfeeding. Patients were genetically unrelated ethnic Han Chinese women from Nanjing City and its surrounding regions. All subjects had complete clinical data or follow-up information as shown in Table 1.

**Table 1:** Patient characteristics of the population in the present study

**Characteristic**	**n (%)/median (range)**
Age (years)	61.2 years (26.0 - 82.5)
BMI (index)	26.3 (17.2 - 36.7)
Histology, n = 108	
HG-SOC	48(44.4 %)
LG-SOC	24(22.2 %)
Serous cystadenoma	20(18.5 %)
Normal ovary	16(14.8 %)
FIGO stage, n = 72	
I	13(18.0 %)
II	7(9.7 %)
III	37(51.4 %)
IV	15(20.8 %)

HG-SOC, High-grade serous ovarian cancer.

LG-SOC, Low-grade serous ovarian cancer.

BMI, Body mass index.

FIGO, International federation of gynecology and obstetrics.

Tumor and matched non-tumors ovarian tissues were collected from 24 LG-SOCs, 48 HG-SOC, 20 serous cystadenomas, and 16 non-tumor control ovaries. The median age of patients was 55 years (38 to 77 years). Samples were preserved in RNAlater reagent (QIAGEN) and stored at −80°C until further analysis. Tumor adjacent non-tumor tissue is defined as at least 1 cm far away from cancer tissue boundary and pathologically confirmed non-tumor. Normal samples were pathologically excluded tumor in ovary. All resection specimens were reviewed by two pathologists from JICR (XY. Xu and N. Hou). Tumor tissues available for 8-OHdG level, mRNA and protein expression assays were all tissue slides confirmed for quality control. The study was approved by the Local Ethic Committee of the Jiangsu Cancer Institute, and each patient provided a written consent.

### 8-OHdG levels in genomic DNA of tissue

Based on the salting out method, DNA extraction from normal ovary, tumor, and adjacent non-tumor tissues (10 mL, with EDTA added to prevent coagulation) was performed within one hour after collection. Eppendorf BioPhotometer Plus (Eppendorf, North America) was used to check the purity of the DNA sample through OD260 nm/OD280 nm, and OD260 nm/OD230 nm. After dissolving into 135 μL water, DNA (200 μg) was mixed with sodium acetate (15 μL, 200 mM) and nuclease P1 (15 μL, 6 units, Sigma, USA) and incubated at 37°C for 30 min. After another 30 min at 37°C, Tris–HCl buffer (15 μL, 1 M, pH 7.4) and alkaline phosphatase (7 μL, 2 units, TAKARA, Shiga, Japan) were added. An ELISA kit (Highly Sensitive 8-OHdG Check, JaICA, Fukuroi, Shizuoka, Japan) was used to measure 8-OHdG according to protocol. Results were converted to 8-OHdG/10^6^ dG according to a previously reported method [23].

### Immunohistochemical staining for hOGG1 and p53 in EOC

Polyclonal anti-hOGG1 (1:1000, Novus Biologicals, NB 100–106) and monoclonal anti-p53 (1:100, Novocastra, DO-7) antibodies were obtained commercially. Mouse IgG prepared against glyceraldehyde 3-phosphate dehydrogenase (GAPDH) was obtained from Chemicon International, Inc. (Temecula, CA). The hOGG1 immunostaining results were divided semiquantitatively into four groups: 0 = no immunostaining present; 1 = weak immunostaining (< 10% positive staining cells); 2 = moderate immunostaining (10-50% positive staining cells); 3 = strong immunostaining (> 50% positive staining cells). We define “0”, “1” as “negative” and “2”, “3” as “positive” [24]. Immunohistochemical analysis of paraffin-embedded section (IHC-P) staining of p53 was assessed semi-quantitatively using a previously reported standard [17]. Ten percent of neoplastic cells were used as a cutoff from negative expression for p53 immunostaining. Staining evaluation was performed by two independent pathologists.

### hOGG1 expression through protein immunoblot

In accordance with the instructions for the total protein extraction kit, total protein was extracted from 100 mg specimens. Protein concentrations were assayed via the Bradford method, and specimens were adjusted to the same protein concentration, packaged, and preserved at −70°C for later use. With a prestained marker serving as an index, required gels were selected after polyacrylamide gel electrophoresis was performed, and a nitrocellulose filter was used for the transfer print. The primary antibody concentration was 1:1000 and the secondary antibody was 1:2000. Using alkaline phosphatase coloration, the protein hybridization band was scanned with a GIS-2020 digital image analysis system and the absorbance value was assayed. The ratios of hOGG1 and GAPDH were calculated for the semi-quantitative analysis.

### *hOGG1* mRNA expression from EOC tissue

Total RNA from tissue samples was extracted using Trizol (Invitrogen, Carlsbad, CA) and ethanol precipitation, according to the instructions of the manufacturer. Primers and probes for *hOGG1* mRNAs were 5′-TGACACCTCACCTCACCCAC-3′, 5′-CACTGTCTTCCGCAAGTTCAC −3′, and TaqMan 5′ (6-FAM)-ACCCTGGTCCGAGGTGTCCCTGAG-(TAMRA-Q) 3′. Primers for β-actin mRNA were 5′- CCAACCGCGAGAAGATGA −3′ and 5′- CCAGAGGCGTACAGGGATAG −3′. Analysis of *hOGG1* expression was carried out via RT-PCR. Quantitative detection of *hOGG1* mRNA was performed using the Quantitect Multiplex RT-PCR Kit (QIAGEN), following the instructions of the manufacturer. Reaction was performed in a 25 μL solution containing 1x Master mix, 0.25 μL RT mix, 0.4 μM of each primer pair, 0.2 μM probe, and 250 ng RNA. Reaction mixture was reversely transcribed at 50°C for 20 min, and heated at 95°C for 15 min before being amplified, using 40 cycles of thermo cycling, with each cycle at 94°C for 45 s and at 60°C for 1.15 min. Samples were normalized based on β-actin mRNA content. Amplification of β-actin was also performed using the kit form QIAGEN. PCR was performed in a 20 μL solution containing 1x SYBR RT Master Mix buffer, 0.2 μL Quantitect RT mix, 0.4 μM of each primer, and 200 ng RNA. PCR occurred following reverse transcription at 50°C for 20 min and heating at 95°C for 15 min through 35 cycles of amplification, with each cycle at 94°C for 15 s, 48°C for 20 s, and 72°C for 30 s. Tissue samples with more than 150 copies of β-actin mRNA were used for *hOGG1* mRNA analyzing (Additional file 1: Figure S1, Additional file 2: Figure S2 and Additional file 3: Figure S3).

### Statistical analysis

All statistical analyses were carried out using the statistical program SPSS version 13.0. Descriptive statistical values included mean ± SD values for continuous data and percentages for categorical data. Differences in protein and mRNA levels as well as in 8-OHdG concentrations among subgroups were evaluated using the student *t*-test, and *t*-test was used for categorical variables such as immunostaining results. In all cases, a p value of less than 0.05 was considered as statistically significant. The Cox proportional hazards model was used to assess the association between overall survival and clinicopathological characteristics including the 8-OHdG level. The Kaplan–Meier estimate was calculated by the log-rank test for overall survival and progression-free survival, values stratified by 8-OHdG alteration.

## Results

### 8-OHdG levels increased in HG-SOC compared to tumor-adjacent normal tissue

**Figure 1:**
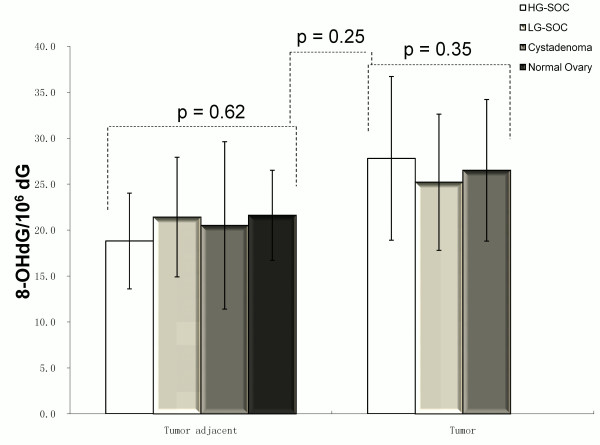
8-OHdG DNA levels in tumors and matched adjacent normal tissues of HG-SOC, LG-SOC, serous cystadenoma, and normal ovaries (mean ± SEM).

**Figure 2:**
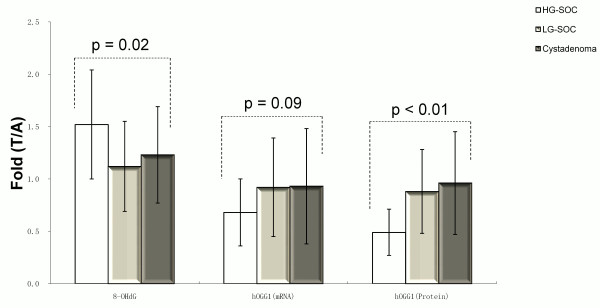
**8-OHdG, *hOGG1 *** **mRNA and protein expression alteration in tumors and matched adjacent normal tissues of HG-SOC, LG-SOC, serous cystadenoma, and normal ovaries (mean ± SEM) (T, tumor tissue; A, adjacent normal tissue).**

8-OHdG levels in tissue DNA were not statistically different in HG-SOC (27.8 ± 8.98/10^6^dG), LG-SOC (25.2 ± 7.4/10^6^dG), and benign serous cystadenoma (26.5 ± 7.7/10^6^dG; Figure 1, p = 0.35). There was no difference in a parallel investigation of tumor-adjacent normal tissue in HG-SOC (18.8 ± 5.2/10^6^dG), LG-SOC (21.4 ± 6.5/10^6^dG), benign serous cystadenoma (20.5 ± 9.1/10^6^dG) and non-tumor ovary (21.6 ± 4.9/10^6^dG; Figure 1, p = 0.62). Higher fold of 8-OHdG expression in the tumor compared with that in the normal tissue adjacent to carcinoma was found in HG-SOC, but not in LG-SOC or serous cystadenoma (Figure 2, p = 0.02).

### *hOGG1* gene expression was lower in HG-SOC compared to tumor-adjacent normal tissue

#### *hOGG1* mRNA expression

**Figure 3:**
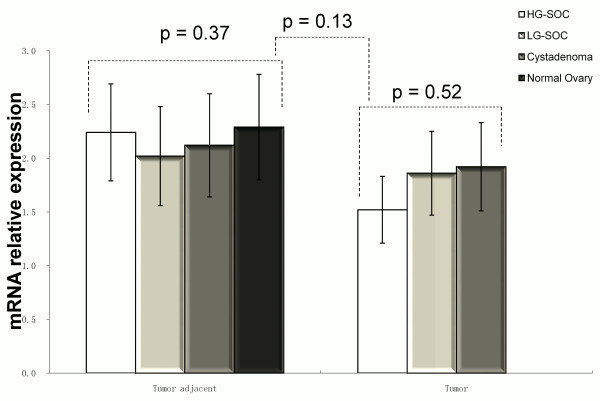
***hOGG1 *** **mRNA levels in tumors and matched adjacent normal tissues of HG-SOC, LG-SOC, serous cystadenoma, and normal ovaries (mean ± SEM).**

There was no difference of *hOGG1* mRNA levels among HG-SOC, LG-SOC, and serous cystadenoma using RT-PCR analysis (p = 0.52, Figure 3). No difference was noted among the tumor-adjacent normal tissues from HG-SOC, LG-SOC, serous cystadenoma, and normal control (p = 0.37, Figure 3). *hOGG1* mRNA level in tumors decreased compared with tumor-adjacent normal tissues in HG-SOC, but there was no statistical significance (p = 0.09, Figure 2).

### hOGG1 protein immunoblot

**Figure 4:**
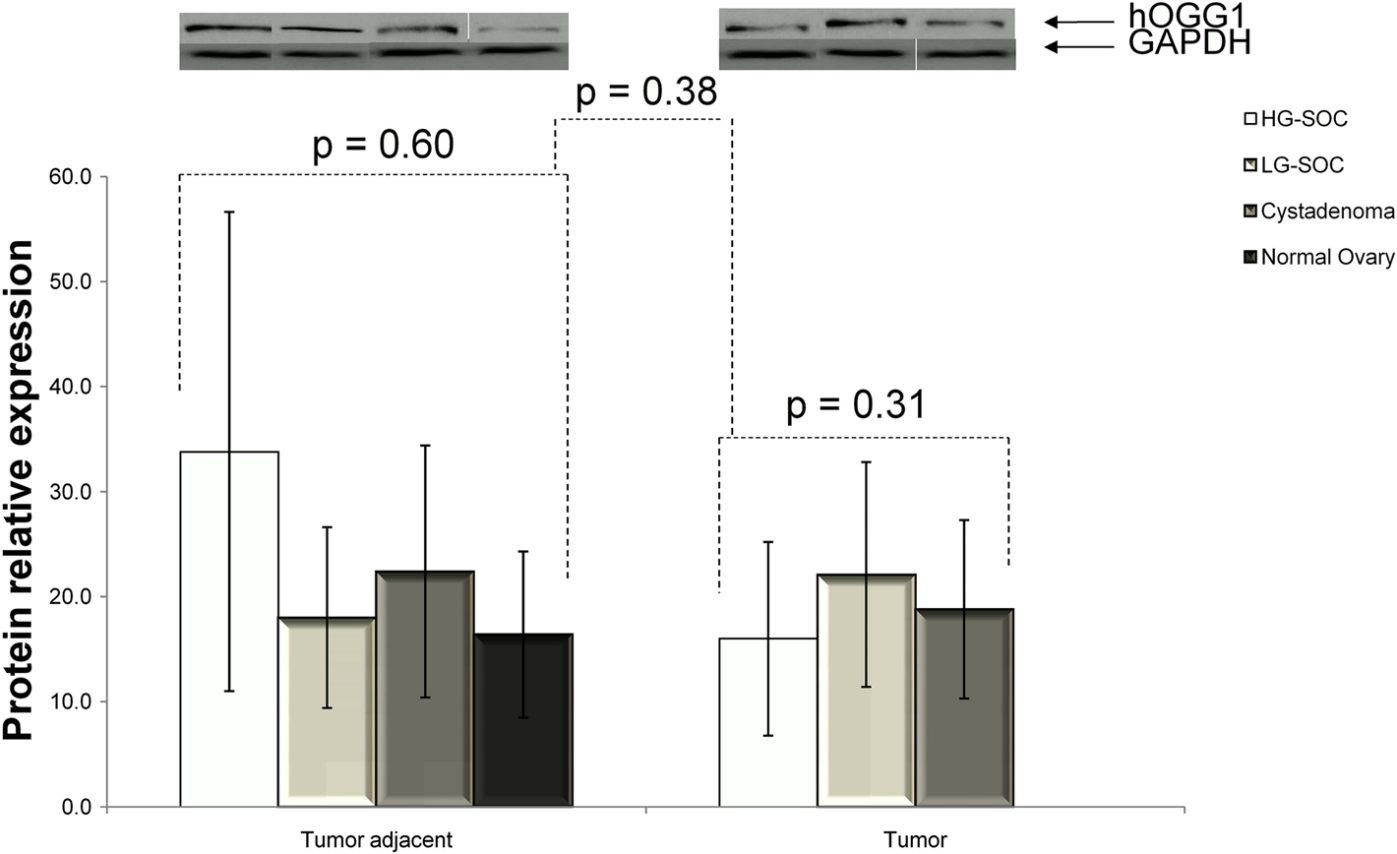
hOGG1 protein expression in tumors and matched adjacent normal tissues of HG-SOC, LG-SOC, serous cystadenoma, and normal ovaries (mean ± SEM). hOGG1 and GAPDH are apparent at 39 kDA and 37 kDa, respectively.

There was no difference of hOGG1 protein expression in HG-SOC, LG-SOC, and serous cystadenoma (p = 0.31, Figure 4), as well as among tumor-adjacent normal tissues, including HG-SOC, LG-SOC, serous cystadenoma, and normal control (p = 0.60, Figure 4). hOGG1 protein expression level in tumors decreased compared with tumor-adjacent normal tissues and the degree was more remarkable in HG-SOC than in LG-SOC and serous cystadenoma (p < 0.01, Figure 2).

### P53 positive-staining is associated with lower hOGG1 expression in HG-SOC

**Figure 5:**
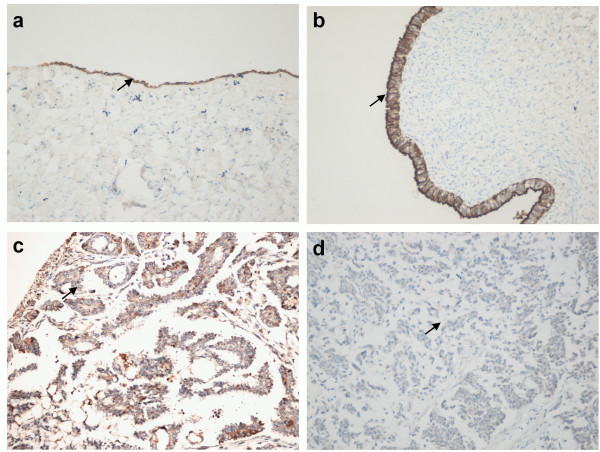
**IHC-P analysis of hOGG1 protein expression in serous ovarian cancer: (A)** Normal ovarian tissue treated with anti-hOGG1 antibody, biotinylated anti-rabbit IgG secondary antibody, and avidin-biotin-peroxidase complex, and counterstained with p-dimethylaminobenzaldehyde reagent (magnification 100 x); **(B)** serous cystadenoma with positive staining (magnification 200 x); **(C)** LG-SOC tissue with moderate positive staining (magnification 200 x); and **(D)** HG-SOC with negative staining (magnification 200 x). Black arrow pointed at the immunostained epithelial cells.

Negative IHC-P staining of hOGG1 was more frequently in HG-SOC than in LG-SOC and serous cystadenoma; however, no statistical significance (p = 0.19, Figure 5) was noted.

Compared with that of LG-SOC, the positive-staining rate of p53 in HG-SOC tissues was significantly higher (75.0% vs. 20.8%, p < 0.01). P53 positive-staining was associated with hOGG1 lower expression in HG-SOC (p = 0.04) but not in LG-SOC (p = 0.55, Table 2).

**Table 2:** p53 correlated with hOGG1 expression in ovarian cancer

**Subgroup**	**p53 immunostaining**	**hOGG1(+)**	**hOGG1(−)**	**p value**
LG-SOC	(+)	4(16.7 %)	1(4.2 %)	0.55
	(−)	15(62.5 %)	4(16.7 %)	
HG-SOC	(+)	10(20.8 %)	26(54.2 %)	0.04
	(−)	8(16.7 %)	4(8.3 %)	

### Increasing 8-OHdG level is associated with poor prognosis in serous ovarian cancer

**Figure 6:**
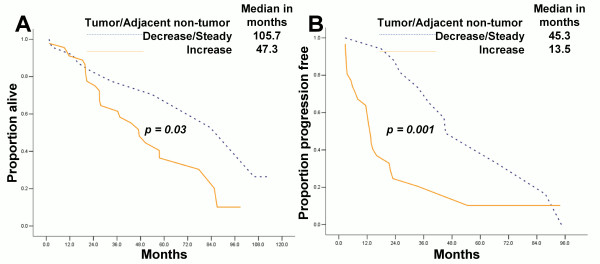
**Increasing 8-OHdG expression in tumor DNA is associated with poor prognosis of serous ovarian cancer patients (N** = **72). (A)** 8-OHdG level associated with overall survival; **(B)** 8-OHdG level associated with progression-free survival.

The Cox proportional hazards analysis revealed that increasing 8-OHdG level independently associated with overall survival in serous ovarian cancer (p < 0.01, Table 3). We further found that increasing 8-OHdG level (p = 0.03 and p = 0.001, Figure 6), but not hOGG1 expression alteration (Additional file 4: Figure S4), in tumor was associated with shorter overall and progression free survivals.

**Table 3:** **8-OHdG alteration associated with overall survival of serous ovarian cancer patients (N** = **72)**

**Characteristic**	**Univariate analysis**		**p value**	**Multivariate analysis**		**p value**
	**HR**	**95 % CI**		**HR**	**95 % CI**	
FIGO stage						
I	1.00	Reference		1.00	Reference	
II	1.42	0.68-6.25		1.21	0.43-8.02	
III	6.78	1.66-18.41		2.25	1.24-6.08	
IV	9.43	3.61-27.87	<0.01	3.28	1.47-9.54	<0.01
Grade	4.12	1.35-12.69	<0.01	2.57	1.62-3.45	<0.01
Suboptimal CRS	2.16	1.25-3.85	<0.01	1.65	1.19-2.55	0.02
Ascites	1.47	1.26-3.04	0.01	1.23	1.08-2.36	0.04
Nadir CA-125 level	1.02	1.01-1.04	<0.01	1.01	1.00-1.03	<0.01
p53 positive	1.30	1.09-4.39	0.02	1.13	0.96-2.41	0.10
8-OHdG (T/A)	1.35	1.13-3.42	<0.01	1.18	1.01-2.07	<0.01
hOGG1 protein (T/A)	0.84	0.78-1.06	0.54	0.89	0. 88–1.02	0.65
Age	1.01	1.00-1.01	0.04	1.00	0.98-1.01	0.21

HR, Hazard ratio; CI, Confidence interval; CRS, Cytoreductive surgery. T/A, Tumor/Adjacent non-tumor tissue.

## Discussion

EOC is a serious health problem among the female population worldwide. Type II subtype of this tumor is responsible for 90% of deaths associated with this disease, and it has different tumorigenesis with type I patients. HG-SOC, the major subtype of type II ovarian cancer, is associated with p53 mutation and decreased ability of DNA damage repair [25,26]. We observed in HG-SOC, but not in LG-SOC and serous cystadenoma, that the average 8-OHdG/10^6^dG level was significantly higher in tumor DNA, and protein expression of hOGG1 was remarkably decreased in the tumors, compared with those in matched non-tumor tissues. The status of p53 immunostaining was also associated with hOGG1 expression in HG-SOC.

Base excision repair, as one of the major DNA damage repair systems, includes several enzymes with cooperative and compensatory enzymatic activities and further associated with the susceptibility of tumor developments. It’s difficult for intuitively estimating the weight of one enzyme dysfunction as an alternative to the overall repair ability. Our results show that no difference existed between *hOGG1* mRNA and protein expressions among HG-SOC, LG-SOC, and serous cystadenoma, as well as between tumor-adjacent tissues and normal ovaries. Previous studies led to conflicting conclusions on the relationship between *hOGG1* expression and cancer risk. Li et al. [27] found that normal breast tissues from cancer patients had a significantly higher level of oxidative DNA damage. The elevated level of 8-oxo-dG in cancer patients was not related to deficiency of the *hOGG1* expression. Other studies reported that *hOGG1* gene expression was markedly suppressed in up to 38% of head and neck squamous cell carcinomas [28,29]. In the present research, we used paired specimens from tumor and adjacent non-tumor tissue to eliminate individual differences. We found that 8-OHdG level and hOGG1 protein expression were associated with the grade of SOC.

Serous carcinoma was reported to be correlated with p53 mutation [30]. Diminished base excision repair was also reported in p53 mutant and p53-null cells [31]. These cells exhibited very low activity of DNA β-polymerase, an enzyme required for repair. Immunohistochemical positive status of p53 is commonly thought to correlate closely with mutation status and lost the activity as the tumor suppresses the gene. We found that p53 positive immunostaining is related to decreasing protein level of hOGG1 in HG-SOC. Does p53 dysfunction induce a decrease in hOGG1 expression, or vice versa? Hence, further studies are needed to test the causality relationship between p53 mutation and hOGG1 expression on the carcinogenesis of HG-SOC.

This study also has several drawbacks. Firstly, tumor tissues and adjacent normal tissues were mostly sampled from surgical biopsies. 8-OHdG level and *hOGG1* expression might have been influenced by neo-adjuvant chemotherapy in several cases, though our experiment had controls. Secondly, in spite of having tissue slide sections for quality control, non-tumor cells such as lymphocytes and fibroblasts may interfere in tumor cells during 8-OHdG and hOGG1 assays, which also affected the accuracy of our results. Thirdly, increase in oxidative damage products and decrease in damage repair ability linked with advancement in age was reported to be associated with age-related diseases [32,33]. Meanwhile, HG-SOC patients were relatively older than LG-SOC cases [34]. Is this a satellite phenomenon that requires serious consideration?

The poly (ADP-ribose) polymerases (PARPs) are a series of enzymes in DNA repair pathways, especially the BER for DNA single-strand breaks repair. Inhibition of PARPs can lead to cell death and genomic instability in DNA repair-defective tumors (like those with BRAC1/2 mutations), what is so called "synthetic lethality". Hooten et al. [35] identified an interaction between two DNA repair proteins, OGG1 and poly (ADP-ribose) polymerase 1 (PARP-1). They provided evidence that cells are more sensitive to PARP inhibitors as a single agent or in combination with a DNA-damaging agent, H_2_O_2_, in the absence of OGG1. These data imply that an identified DNA glycosylase dysfunction or a concurrent *hOGG1*-targeted therapy may possibly help in clinical trials involving patients with BRCA1/2-mutated or not ovarian carcinoma treated with PARP inhibitors [36-39].

In conclusion, we believe that defects in *hOGG1* expression and increase in 8-OHdG levels may have significant influences in the development of HG-SOC. 8-OHdG and *hOGG1* levels may be used as molecular markers to distinguish the two types of SOC. Moreover, specific genetic or regulation abnormalities in different EOC subtypes may help in the development of specific drugs.

## Abbreviations

EOC: Epithelial ovarian cancer; HG-SOC: High-grade serous ovarian carcinoma; LG-SOC: Low-grade serous ovarian carcinoma; hOGG1: Human 8-oxoguanine (8-OHdG) DNA glycosylase.

## Competing interests

All authors declare no conflicts of interest.

## Authors’ contributions

XX, XXC and KJLparticipated in drafting the full manuscript and writing of this manuscript. FD and JN partly participated in clinical study design, coordination and data analysis. YW, WWG, YQZ and CML participated in collecting data, creating figures and tables. WWG contributed by writing specific sections of this manuscript. YW, YQZ and CML provided advice and participated in revising the manuscript. XXC participated in substantial contribution to conception and revising it critically for important intellectual content. All the authors in this manuscript have read and approved the final version.

## Supplementary Material

Additional file 1: Figure S1 Electrophoresis of RT-PCR products of *hOGG1*. Click here for file

Additional file 2: Figure S2 Amplification plot of *hOGG1* mRNA level assay. Click here for file

Additional file 3: Figure S3 Melt curve of *hOGG1* mRNA level assay. Click here for file

Additional file 4: Figure S4 hOGG1 protein expression is not associated with prognosis of serous ovarian cancer patients (N = 72). (A) hOGG1 protein level is not associated with overall survival; (B) hOGG1 protein level is not associated with progression-free survival. Click here for file
